# Contemporary dental tourism: a review of reporting in the UK news media

**DOI:** 10.1038/s41415-025-8330-2

**Published:** 2025-02-28

**Authors:** Janine Doughty, Deborah Moore, Matthew Ellis, Jazz Jago, Prasanthi Ananth, Alexander Montasem, Alexander C. L. Holden, Ilona Johnson

**Affiliations:** 41415363003001 School of Dentistry, University of Liverpool, UK; 41415363003002 Public Health, Policy and Systems, University of Liverpool, UK; 41415363003003 University Hospitals Bristol and Weston NHS Trust, Bristol, UK; 41415363003004 Sydney Dental Hospital, Sydney Local Health District, Australia; University of Sydney School of Dentistry, Sydney, Australia; 41415363003005 Public Health Wales, Wales, UK

## Abstract

**Introduction** The number of people seeking dental tourism increased in recent years and has peaked in popularity with young people following a wave of viral social media content. Dental professionals have expressed their concern about the short- and long-term consequences. This study aimed to explore the contemporary United Kingdom (UK) media narrative toward dental tourism.

**Methods** Newspaper articles were identified using the LexisNexis database. The ten most popular newspapers in the UK were used for the search strategy. Data were analysed using framework analysis. The findings are presented as descriptive and analytical themes.

**Findings** The search strategy identified 201 newspaper articles related to dental tourism. A total of 131 articles were included in the analysis. Five key themes were identified. The themes included: push and pull factors reported to lead to seeking dentistry abroad; patient-reported outcomes and experiences; warnings from dental professionals; amplifying social media hype; and media shaming and stigmatising.

**Conclusions** Social media viral health trends were a means of distributing health (dis/mis)information. The perspectives of social media were amplified by the UK press. Tabloids often stigmatised people who had dentistry abroad.

## Key points

- People are commonly reported to be seeking dentistry abroad to address issues of low self-esteem or confidence related to their oral health, or due to issues of access and affordability regarding routine dental care in the UK.
- Journalism examined in this study failed to recognise and acknowledge that the phenomenon of dental tourism should be broken into two separate components: 1) dental tourism that promotes access to routine dental care; and 2) dental tourism for purely cosmetic reasons.
- Journalistic approaches to dental tourism in tabloid newspapers commonly amplified polarised social media rhetoric, shaming or ridiculing people who have chosen to seek cosmetic dental procedures outside of the UK, especially those who have suffered post-operative complications of cosmetic dental procedures.
- Consumers require more guidance to support them in accessing quality dental care outside of the UK and compassion from UK dental professionals if there are post-operative complications arising from dental tourism.

## Introduction

In recent years, the practice of international travel for low-cost dental care has increased in popularity. The phenomenon is referred to as dental tourism. Dental tourism is affecting many high-income countries and commonly occurs along regional, as opposed to global, pathways. For example, the combination of a holiday combined with cut-price dental treatment has led Turkey, Hungary and Poland to emerge as key players in the British dental tourism industry; people in the United States seek inexpensive dental care in Argentina, Costa Rica or Peru; and people in Australia travel for low-cost dentistry in Indonesia or Thailand.^1^^,^^2^

In the United Kingdom (UK), dental tourism is on the increase. In 2014, 48,000 people sought dentistry outside of the UK. In 2016, the number had increased to 144,000.^3^ Motivations for medical tourism can be categorised into pushing and pulling factors, for example, quality, efficiency, holidays and hospital reputation exemplify pulling factors.^4^ Other reasons include reducing treatment timescale or increasing the variety of treatment options on offer.^1^ Conversely, pushing factors that drive patients to seek care outside of the UK include high cost of treatment, long waiting lists and lack of dental care availability. Reportedly, some people travel abroad because of a lack of trust toward National Health Service (NHS) dentists, difficulty registering with an NHS dentist and ‘amateurish' results of dental treatment in the UK.^4^

Dental professionals have expressed their concern about the recent rise in young people opting for dentistry abroad. The British Dental Association (BDA) surveyed 1,000 dentists who described the adverse health consequences associated with dental tourism. Almost all respondents to the survey reported that they had examined patients who had been on dental tourism trips and most (86%) reported treating people suffering consequences after treatment abroad.^5^ Respondents believed that crowns and implant treatments were the most at risk of failure. On average, the cost of remedial dental care in the UK ranged between at least £500 (65%) up to more than £1,000. However, a significant number (20%) of dentists estimated the cost to rectify complications arising from dental tourism as in excess of £5,000.^5^ In response to the dental tourism trend, the BDA has issued recommendations for more awareness raising of the risks, including proactive campaigns to inform the public.^6^ Further, concerns have been raised about child patients being offered invasive restorative treatment for minor aesthetic concerns as a ‘freebie' to their parent's course of treatment.^7^

The Department of Health has previously indicated NHS responsibility to provide emergency care, but not remedial care (eg elective revision), for cosmetic procedures provided outside of the UK.^8^ Asher and colleagues^9^ have queried whether NHS Trusts could heighten their presence in the international market as providers of private services. They suggest this may eliminate medical-ethical concerns associated with substandard cosmetic tourism.

In the UK, controversy has been escalating around the trend for young adults to seek dental transformations and share their experiences on social media platforms such as Instagram and TikTok. The negative impacts of dental tourism were brought to the attention of the wider public consciousness by a BBC documentary entitled *Turkey teeth: are they worth it?* which was released in July 2022.^10^ This created a wave of media interest in the topic.

In the UK, newspapers account for almost 40% of the nation's source of news, and attitudes toward the quality, accuracy, impartiality and trustworthiness have remained steady in recent years.^11^ Public discourse and the framing of public health problems in national newspapers both shapes and reflects public opinion.^12^^,^^13^ Newspapers can also be a tool for health advocacy. However, different newspapers have different stances on public and political issues and different readerships.^14^ Therefore, analysing newspaper content on dental tourism could provide valuable insights into the public opinion toward dental tourism. Thus, the two-fold aim of this study was to understand what the key topics or issues relating to dental tourism in the UK news media are, and how the UK news media frames dental tourism.

## Methods

### Ethics statement

The study synthesised and interpreted secondary data in the form of newspaper articles that were already published and readily available in the public domain. No human participants were involved in the study. Therefore, formal processes of ethical approval were not required.

### Search strategy

Newspaper articles were identified using the LexisNexis database.^15^ LexisNexis is a data analytics company; its databases are accessed through online portals, including portals for computer-assisted legal research, newspaper search and consumer information. The ten most popular newspapers in the UK were used for the search strategy: popularity was based the Audit Bureau of Circulation data on the circulation of both daily and Sunday print publications, as well as access of online articles. Popularity was based on readership of each newspaper. 

A pilot search was used to identify key words and to decide the date parameters for inclusion. The pilot search included hand searching 50 news articles using a search engine and a search for academic articles using the search string ‘dental AND tourism'. This investigative search revealed that terminology used by the media differs from that used by the dental community. For example, newspaper articles tended to use phrases such as ‘dentistry overseas' or ‘dentistry abroad'. Contrastingly, dental articles or newspaper articles offering advice from dental professionals commonly used the phrase ‘dental tourism'. The full search strategy is presented in Table 1.

**Table 1:** Search strategy

Search strategy	‘Turkey teeth' OR ‘Dental vacations' OR ‘Overseas dental treatment' OR ‘Dental travel' OR ‘Dental holidays' OR ‘Dental trips abroad' OR ‘Dental tourism' OR ‘International dental care' OR ‘Dental procedures overseas' OR ‘Dental treatment options overseas'
Publication location	Europe Publication Location: United Kingdom of Great Britain & Northern Ireland
Content type	News
Publication language	English
Timeline	1 January 2018 to 31 May 2023
Publication type	Newspapers
Publication name	thesun.co.uk, MailOnline, *The Sun* (England), *Metro* (UK), *The Daily Mail* and *Mail on Sunday* (London), *The Daily Telegraph* (London), *Daily Star, The Times* (London), *Scottish Daily Mail, The Guardian* (London), *Daily Mirror, The Sunday Times* (London), *The Sunday Express,* mirror.co.uk, *The Express*, standard.co.uk, *The Evening Standard* (London), *The Observer* (London)

### Inclusion and exclusion criteria

Authors have identified media interest in dental tourism as first emerging around two decades ago.^16^ However, this review article specifically focused on contemporary narratives and thus used 2018 as the cut-off point. The reason for this decision is that, in 2018, UK reality television celebrities began to disclose having had dental treatment abroad. This has been heralded in many newspaper articles as a defining catalyst for the most recent wave of dental tourism which has uniquely impacted on younger people and social media.

Through a process of independent review and collaborative discussion three reviewers (JD, PA, JJ) identified which papers met the inclusion and exclusion criteria. Articles from tabloid and broadsheet newspapers were included. Tabloids are newspapers which are typically sensationalist and report more celebrity material. Broadsheets are perceived to be more intellectual in content.

Advertorials were excluded from the analysis, as were articles which pertained to other healthcare procedures performed outside of the UK. Articles which referred to someone as having ‘Turkey teeth' but where the topic of the article was not related to dental tourism were excluded.

## Data extraction

Data were extracted into an Excel spreadsheet. Articles were mapped to identify the newspaper name, type (tabloid or broadsheet), the annual frequency of articles published on dental tourism each year between 2018-2023, and the central protagonist (dental professionals, patients, journalists, dental organisations, others, or multiple). Critical appraisal was not undertaken due to the non-academic nature of the articles.

Full-text papers were read by JJ, JD, and PA. JJ, JD, PA and DM met twice to discuss the codes and the themes in depth. All authors reviewed and refined the final themes. All textual components of the articles, including journalists' narratives and quotations, were used as data and analysed using framework analysis. The following stages of the framework approach were undertaken: familiarisation; coding; developing and applying an analytical framework; and charting and interpreting the data.^17^ Inductive coding was carried out to identify descriptive and analytical concepts within the data. Initial codes were created by JD, JJ, PA and DM after reviewing the first 50 articles. These codes were then applied by JD, JJ and PA to the remaining 81 articles. Where new concepts were identified that were not adequately captured by other codes, new ones were created. The codes were reviewed and refined by JD, JJ, PA and DM to merge similar concepts, separate different concepts and refine the code descriptor to concisely reflect the intended meaning. The codes were then reviewed by JD, JJ, PA and DM to organise them into overarching analytical themes to answer the research questions. The findings are presented as themes. Finally, these themes were then assessed by the full authorship team for their relevance and appropriateness to answering the research questions.

## Results

**Fig. 1:**
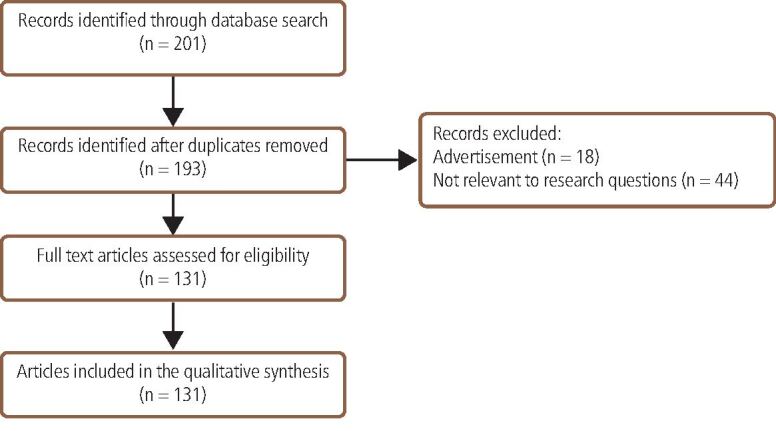
Flow diagram of included and excluded articles

The search strategy identified 201 newspaper articles related to dental tourism. A total of 131 articles were included in the analysis (Fig. 1). The remainder were excluded because they were advertisements, were not related to the research question, or were duplicated articles published both in paper and online versions of the same newspaper.

Most articles were published in 2022 and 2023 (Table 2). The 2022 and 2023 peaks in article numbers coincided with the publication of dental contract reforms, the emergence of the TikTok trend of #TurkeyTeeth and the release of the investigative television show ‘*Turkey teeth: bargain smiles or big mistake'* in July 2022.

**Table 2:** Number of articles published by year

**Year**	**Number of articles published**
2018	0
2019	1
2020	12
2021	0
2022	59
2023	59
**Total**	**131**

Ten articles (7.6%) were published in broadsheet newspapers. The remaining articles (92.4%) were published in tabloid newspapers, and of these, 106 (80.9%) were published in either *The Sun* or a newspaper linked to *The Mail* (eg *Daily Mail,* MailOnline etc.) (Table 3).

**Table 3:** Distribution of articles across broadsheet and tabloid newspapers

	**Paper**	**No. of articles**
Broadsheet	*The Guardian*	3
	*The Times* and *The Sunday Times*	2
	*Daily Telegraph*	5
Tabloid	*The Sun*	83
	*The Metro*	1
	*Scottish Daily Mail/Mail Online/Mail on Sunday/Daily Mail*	23
	*Evening Standard*	1
	*Daily Mirror/Mirror*	6
	*Daily Star*	7

Five key themes were identified from analysis of the newspaper articles. The themes included: push and pull factors reported to lead to seeking dentistry abroad; patient-reported outcomes and experiences; warnings from dental professionals; amplifying social media hype; and media shaming and stigmatising.

### Motivators: push and pull factors leading to seeking dentistry abroad

Pull factors are those that draw people to another country for dentistry. Push factors are those that encourage people to leave their country of residence. In this study, we found that pull factors included celebrity influence, treatment affordability, and seeking a quick fix to improve appearance and self-esteem. Push factors included perceptions of high costs of dental treatment and difficulties accessing dental care.

#### Self-esteem and social signalling

The newspaper articles commonly highlighted celebrities who had sought dentistry abroad. Celebrity status and television shows, specifically celebrities featured on reality shows such as Love Island, were commonly mentioned. All references were from tabloid newspapers:

- ‘Love Island winner…who travelled to Turkey for ten crowns before going on the reality show in 2018, said he had not realised “it was quite as invasive as it was”. He added: “my mum used to be a dental nurse so I know how expensive it is to get your teeth done. I knew it would be about £10,000 to £15,000 easily in England. So I thought I'd rather just go to Turkey get a bit of sun, have a laugh”' (celebrity, article 2).

Motivators for seeking dental treatment abroad also included low self-esteem or confidence related to the appearance of teeth and the lure of a quick fix to improve appearance and boost confidence:

- ‘I'd read about the Turkey teeth quick fix and went online watching influencers rave about their new cheap smile and a sun-filled holiday. I was won over and instantly booked a trip to Turkey to have my teeth fixed and to grab a much-needed break. I arrived in July 2018 and saw the clinic's dentist, he took an x-ray and told me I needed crowns but no more than that…within hours I was injected with painkillers' (patient, article 77).

Some articles described people with multiple vulnerabilities including issues with self-esteem which made them more susceptible to being leveraged by predatory marketing. One article contrasts the marketing for a company who provide cosmetic dental treatment in Turkey (which appeared on the London Underground and on buses) with how UK practitioners are expected to approach marketing, noting that the General Medical Council guidance for practitioners offering cosmetic treatment insists that ‘marketing must be responsible' and should not, ‘minimise or trivialise the risks of interventions'.^18^ In one article, a UK dentist says:

- ‘It's companies that are trying to make money on impressionable and often vulnerable people, who are unaware of the ramifications of what they are getting themselves into. I'm not allowed to advertise like that to the under 18s yet you have a treatment there which isn't general dentistry, it's not your six month check-up…this is very aggressive' (dentist, article 4).

#### Affordability and difficulties accessing dental services in the UK

A very common recurring theme of articles was highlighting the significantly lower cost of dental and medical cosmetic procedures abroad, especially Turkey, compared to similar treatment in the UK. Cosmetic treatments that many people might have previously believed was beyond their financial means were now accessible. Patients commonly described choosing dentistry outside of the UK because of cost-saving factors, rather than perceptions of higher-quality care:

- ‘Cosmetic work in Turkey comes cheap. Incredibly cheap, generally a third of what you'd pay at a UK clinic, sometimes even less. A new nose for 2,500. A full set of “Turkey teeth”, those dazzling, perfect pearly whites that are suddenly everywhere, starts at 3,200…by comparison, rhinoplasty (a nose job) in the UK starts at around 6,200…a new set of teeth at least 12,000' (journalist, article 122)

Several articles, especially those covering the joint BDA-BBC documentary *Disappearing dentists*, highlighted that patients have been unable to unable to gain access to an NHS dentist after calling dozens of dental practices.^19^ The narrative described how people who were unable to afford private dentistry in the UK had to decide whether to have no treatment, perform DIY (do-it-yourself) dentistry, or travel abroad:

- ‘After trying and failing to get an NHS dentist appointment in Britain, recent research found that 91% of UK dental practices are refusing to take new patients - “getting my teeth fixed abroad seemed like a logical option”' (journalist and patient, article 31).

Although most articles told the perspective of a patient or of the journalist, and were often sensationalist in their slant, three articles did contain statements from the BDA, offering more context to the crisis of NHS dental access:

- ‘Unless the government invest loads in the NHS, so everyone can have an NHS dentist, people are going to be in the horrendous position of having to go abroad' (BDA board member and dental practice owner, article 31).

### Patient-reported outcomes and experiences

Many people who had, had dentistry abroad explained that ‘it was worth it', accepting the trade-off of dental health for cosmetic improvements. Some patients accepted dental pain and discomfort in exchange for the improvements to their confidence, self-esteem and self-assessed attractiveness:

- ‘All in all, absolutely mint, we're made up, couldn't be happier. Highly recommend, it doesn't half change your face. Eating is difficult though, it's very sensitive to cold so it's something to get used to a few days afterwards. It's good for the diet because you do not eat whatsoever, it's difficult to eat. Get used to drinking through a straw' (husband and wife who have been abroad for dental care, article 102)
- ‘JAW THING: I broke down in tears when I saw my shaved-off Turkey teeth and had to live on cake and omelettes but now I love them' (headline, article 12)
- ‘TOOTH OF THE MATTER: I got Turkey teeth and I don't care if I regret them in ten years I think they look so good' (headline, article 29).

Several articles featured people describing diet modifications, such as soft diet and drinking through a straw, to mitigate post-procedural discomfort. People who had bad experiences with dental procedures performed abroad described issues such as ‘dead stumps', abscesses and pain. Words commonly used to describe the impact of cosmetic dental treatment undertaken abroad included ‘pain', ‘aggressive', ‘invasive', ‘complications', ‘problems' and ‘infection'. Individuals described irreversible physical harm and ongoing anxiety related to both their dental health and the costs of remedial care.

Confused about the procedures they'd had, people felt misinformed about the implications of the cosmetic treatment. People described mental and physical harm as a result of dental tourism abroad. A few articles described people who had suffered harm following dental tourism and now used their social media influence as a vehicle to deter others from doing the same. Influencers presented their experiences in different ways; some described themselves as victims of poor-quality dental care and confusion around what procedures they were consenting to, while others explained that, although they were aware of the risks, the improved aesthetics were worth it. Others tried to discourage followers from following in their footsteps and saw their role as a health advocate:

- ‘You've done a brave thing…to warn people knowing you'll likely get hate. Fair play spreading awareness. This is a new trend among young people and it's going to be a hard pill to swallow for the unlucky ones' (social media commenter, article 14)
- ‘UH OH: I got Turkey teeth but it was a big mistake. I've now got a lisp and can't close my mouth properly, please don't do it' (headline, article 34).

### Warnings from dental professionals

The destructive dental transformations described in the articles were at odds with minimally invasive dentistry culture in the UK. The articles that described the perspectives of dental professionals had a paternalistic element, with little recognition for the strong motivators/drivers for patients to seek dentistry abroad. Dental professionals quoted in the articles advised that cheap procedures in Turkey may infer inferior-quality treatment and that people can expect to pay similar prices to UK dental treatment for high-quality dental care. Dental professionals unanimously agreed that in the UK, minimally invasive approaches to dental care, such as orthodontic appliances, whitening and bonding, were the preferred practice to maintain health and improve aesthetics.

The lack of regulatory processes and absence of access to legal redress or follow-on care was a common concern across the articles. A few articles described remedial care as being provided by the NHS, while other private providers described actively avoiding the provision of care for people who'd had dentistry abroad because of fear of liability:

- ‘If I did 20 crowns on a 21-year-old for the purpose of improving the colour, I would have my licence revoked, I would be struck off […] at the point you inherit that patient and do any work, that's when the problems really start and that's when the UK dentist becomes liable. A risk we cannot take' (director of a dental clinic in Liverpool, article 51)
- ‘It's shocking people have no clue what they've done. They talk about veneers - mouldings bonded to the front of a tooth - but in reality, they are crowns, meaning much more aggressive tooth reduction' (NHS dentist, article 10).

### Amplifying social media hype

Newspaper articles commonly built their narrative using a single post lifted from social media, specifically TikTok. This tactic frames social media posts and perspectives as noteworthy and of importance in contemporary thinking about dental tourism. These posts documented journeys, complications and/or enhanced aesthetics. Common among them was the use of the #TurkeyTeeth hashtag. The phrase ‘Turkey teeth' has created a group identity: people who have all been to similar places, to have similar procedures, have had similar experiences on their journey and ultimately, obtained a similar aesthetic outcome, and in the future will go through the same challenges, disappointments and expenditure on remedial care. Social media provided a space for people to encourage others to seek dental tourism, to be seen as attractive and rewarded with likes and followers, or to generate a buzz around the potential complications of dental tourism. Journalists used emotionally charged language, for example, describing the numbers of views garnered by videos about dental tourism and #TurkeyTeeth as ‘whopping'. Conversely, newspapers can drive readers to their articles by using the stories of influencers and using provocative negative comments to engage readers. Both positive and negative information about the medical enhancement procedures make headlines:

- ‘Her video has clearly shocked many, as it has quickly gone viral and has racked up a whopping six million views. It has 397.6k likes, 4,595 comments and 6,968 shares' (article 39).

### Media shaming and stigmatising

People were discredited by journalistic descriptions of unrelated character labels (eg blonde-bombshell, woman bitten by a bat, blonde-haired woman) and referred to as ‘brutally mocked' by online trolls (article 22). The language used by journalists conveys a tilt toward scorn, for example, the repeated use of the word ‘dubbed' in reference to the phenomenon of ‘Turkey teeth' and the use of terms that are comical in reference to teeth, for example, calling them ‘gnashers' which is commonly associated with fake teeth (article 53). Many headlines likened people's appearance to animals or made ridiculing comments about the appearance of their teeth.

Quotations drawn from comments on the featured social media posts ranged from support, encouragement and compliments through to victim blaming, stereotyping, labelling, stigmatising and shaming:

- ‘NOT ALL WHITE: I splashed 3.6k on a set of white Turkey teeth but trolls say I look like a horse and most people think I've been scammed' (headline, article 38).

A dichotomy was apparent - those who responded to full-mouth transformations as a symbol of good taste and others for whom it was perceived as distasteful and to be avoided or ridiculed:

- ‘His teeth were fine before but it's up to him what he spends his money on'; ‘looks like that smile filter'; ‘too white? Just me? I mean, they look good and everything but too white, no?'; ‘Turkey is where poor working class go' (TikTok user comments, article 63)
- ‘NOT WHITE: My man and I jetted to Turkey for new teeth - we love our gnashers but it's REALLY divided opinion' (headline, article 69).

Common denigrations included warnings that influencers would live to regret their choices, recommending minimally invasive approaches as a preferred alternative, and referring to people as vain, fake-looking or foolish.

## Discussion

To our knowledge, this study is the first to explore the UK news media content on dental tourism. The articles included in this review were published after the 2018 celebrity endorsements of cosmetic dental tourism abroad. Most articles (92.4%) included in this study that pertained to dental tourism were reported in tabloid newspapers. Most articles were published in *Daily Mail* publications and *The Sun*; these are the first and fourth most popular newspapers in the UK, respectively. Many articles in this study were distinctly stigmatising of people seeking and suffering from the consequences of dental tourism. Almost all of the articles about post-operative complications were Turkey-centric, primarily because the media were focusing on social media accounts using #TurkeyTeeth, as opposed to Turkey specifically being implicated in providing substandard dentistry. The convenience of collectively placing all overseas dentistry within this category should be acknowledged as both unfair and inappropriate, and yet has become a recognised colloquial term in the zeitgeist for what is otherwise a difficult-to-define and complex social phenomenon.

### Celebrity culture, class and dental treatment

Social media and celebrity status have previously been identified as motivators for patients' choice of dental clinic and their perceptions of what constitutes the ideal ‘Hollywood' smile.^20^^,^^21^ People who feel that they are failing to meet the benchmark for the appearance of oral health may experience low self-esteem and dissatisfaction with their appearance. This creates a desire for improved aesthetics and to feel more confident about oneself, which can be achieved by undergoing cosmetic dental procedures. As a result, some people may be increasingly susceptible to predatory marketing which offers cheap, quick fixes and radical overnight transformations abroad.

In 2018, *Forbes* described one of the fastest growing trends in the beauty industry as ‘the instant fix' - products that offered instant gratification and immediate improvements in appearance.^22^ However, this is at odds with conservative approaches to cosmetic dentistry, which often require more time (eg whitening, orthodontics, composite bonding) and after-care (eg retainers, top-up whitening, polishing) compared with the immediate placement of indirect restorations described in the newspaper articles. Therefore, the professional approach to aesthetic/cosmetic dental treatment in the UK is directly at odds with recognised consumer preferences that demand and expect rapid cosmetic outcomes. Conspicuous consumerism is the overspending on goods or services to display one's wealth and social status.^23^ It has been associated with low social self-esteem in those who identify as being in a higher subjective social class.^24^ The impact ofconspicuous consumerism in cosmetic practice drives those seeking dental enhancement to opt for more, rather than less, adjustment to their appearance. In this circumstance, individuals pursue a smile which they and others perceive to be not only attractive, but also one which signals their means to be able to afford this treatment in the first place.^25^^,^^26^ In this paradigm, teeth are modified to be brighter and whiter than is naturally attainable so that there can be no mistaking cosmetic dental intervention has been undertaken. An interesting evolution to the phenomenon of cosmetic conspicuous consumerism is not only the promotion of the aftereffects, but also the cosmetic journey. Individuals may openly publicise the process of teeth being modified for cosmetic adjustment, with the destructive component of teeth preparation being a very visible stage of this path.

Many tabloid articles had a moralising undertone that used likenesses to animals and disparaging descriptors to mock or undermine people who are experiencing the consequences of dentistry abroad. In other studies, tabloid newspapers have been found to present significantly more stigmatising content related to health conditions when compared to broadsheet publications. Newspaper articles in this study tended to amplify the voices of individual social media influencers and their scathing critics, infrequently providing further information beyond the original text and comments. Further, by subtly discrediting individuals (eg blonde bombshells), journalists legitimised the shaming and ostracising of people who have had cosmetic dentistry abroad, thereby diminishing compassion toward them.

Many articles described divided public views around cosmetic dentistry and unnatural appearances of teeth in terms of their shape and shade. In recent years, there has been extensive and increasing coverage of ‘culture wars' in UK tabloid and broadsheet media. Culture wars are conflicts between groups with different cultural ideals, beliefs or philosophies. There are references to ‘Turkey teeth' being associated with poor or working-class individuals and younger people. The newspaper articles in this study demonstrate culture wars, with people being stigmatised as vain or foolish in articles for the consumption of another group of people. Tabloid newspapers in particular legitimised the shaming and ridiculing of people who are unable to afford or access dentistry in the UK and the harm that has come to them as a result of seeking affordable dentistry abroad.

As negative stories about #TurkeyTeeth have gone viral, the outcome of this could be to dissuade people from having ‘Turkey teeth' if they believe they might suffer the same physical and social ramifications of stigma and shame. We cannot be certain whether social media shaming will stem the tide of young people seeking cosmetic dentistry abroad. The current evidence is at odds. While some authors report that the anticipation of public or private (internal) shame can reduce the likelihood of making risky decisions, others have found that anticipated embarrassment can lead to a search for risk.^27^

### Dental tourism and health policy

The journalism examined in this study fails to recognise and acknowledge that the phenomenon of dental tourism should be broken into two separate components: 1) dental tourism that promotes access to dental care; and 2) dental tourism for purely cosmetic reasons. Where an individual decides to seek dental care abroad, either for sociocultural reasons (ie returning to a home country to have care in a more culturally inclusive setting) or for socioeconomic reasons (ie to be able to access affordable care more easily), there should be greater reflection on whether criticism from either the dental profession or the public is appropriate. Most professional dental organisations (representing the profession as members) tend to take a negative view of patients travelling abroad for care and this perspective often fails to account for sociocultural reasons for seeking care.^28^^,^^29^ There is no objective evidence to show that the majority of patients travelling abroad for care experience exploitation or poor clinical outcomes and little evidence-based reflection on the comparative risks of similar issues occurring while seeking care in one's own country. Much of the anecdotal commentary from the profession where dentists report seeing poor outcomes from overseas dentistry could be equally made of care provided domestically.^30^ One of the comments spotlighted in this study of dental tourism being ‘horrendous' is not commensurate with the reality that for some, travelling abroad for care is not a choice begrudgingly taken.

Many of the contemporary dental tourism news articles included in this review specifically focused on the viral hashtag #TurkeyTeeth and the boom of positive and negative social media interest in the topic. At time of writing, #TurkeyTeeth has 700.8 million views on TikTok and 18.2k Instagram posts. In recent years, social media has become a key area of concern for public health. Indeed, social media is now identified as a commercial determinant of health.^31^ Social media platforms have been criticised for failing to moderate mis/disinformation across a number of areas (racism, sexism etc) and healthcare is no exception.^32^ The discourse presented in the newspapers reflects the dangerously individualised and downstream focus of responsibility attributed to the individual decisions and actions regarding dental healthcare utilisation of users. This has led to some professional attitudes promoting a policy position that would prevent those who have paid for dental work overseas from accessing publicly funded dentistry. While a shallow assessment might find some merit in suggesting that personal decisions to have care aboard should not become a burden on the state, this narrative is problematic, as it fails to account for diaspora returning for care in their home country or for those emigrating. There are no similar calls to prevent those who have extensive or expensive care provided privately at home and who then become unable to self-fund maintenance or remediation of dental treatment from accessing care.

### Strengths and limitations

The strengths of this study are that it gives an understanding of the key issues around dental tourism as presented in the UK press media. The study used a comprehensive search strategy and robust methodology to qualitatively analyse the data and develop the study themes. A limitation of this study is that the data were limited to the ten newspapers with the highest print and online readership. We did not include data from non-newspaper sources, such as the BBC website, or television. Further, we specifically sought newspaper narratives from the past five years to understand contemporary perspectives around dental tourism. This limited the number of articles included in the review and does not reflect insights into earlier news reporting of dental tourism. The date of the search is up to May 2023. Therefore, narratives around dental tourism between search and publication data are not represented in this article and may have changed during this time. A content analysis may have been an alternative approach for the analysis of the data presented in this article. The study is UK-centric and the findings may lack transferability to other cultural contexts.

### Implications for practice

Following on from the findings of this study, we make three key recommendations.

#### Regulation of social media advertising for cosmetic dentistry abroad which may be accessed by under 18s

Last year, following a public consultation, the Committee of Advertising Practice and Broadcast Committee of Advertising Practice introduced restrictions prohibiting cosmetic intervention advertising from being directed at people under the age of 18. The restrictions have been in effect since 25 May 2022. The regulations stipulate that cosmetic interventions must not appear in non-broadcast media directed at under 18s, where under 18s make up over 25% of the audience, and during or adjacent to programmes commissioned for, principally directed at, or likely to appeal particularly to, under 18s.^33^ We recommend similar guidelines for cosmetic dental advertising on social media to safeguard children from being exposed to predatory messaging.

#### Guidance for consumers

The General Dental Council has recently published a document about going abroad for dental care which supports consumers during the decision-making process. However, people who wish to seek dentistry abroad may be vulnerable to misinformation and may not understand the treatment that they are being offered. Guidance at a national level should also provide insight about the difference between cosmetic options (eg the difference between crowns or veneers versus Invisalign and whitening), as well as the advantages and disadvantages of each treatment. The NHS has a treatment abroad checklist which identifies a series of warning signs when seeking healthcare outside of the UK.^34^ A list of reputable overseas dental care providers could be compiled to guide patients toward accessing better-quality care. Further, we are in agreement with BDA recommendations for raising more awareness of the risks, including proactive campaigns to inform the public;^5^ although, we also recognise that professional narratives can be vulnerable to bias. In the absence of national leadership from regulatory or health bodies, this role could be facilitated by joint enterprise from professional groups within dentistry and consumer advocacy groups, aligning with professional associations' missions and roles within the social contract.^35^

#### Compassionate dental care

When people and our patients are making difficult decisions about how to improve their appearance and how to do so within the boundaries of their available resources, it is important to respond with compassion. As a profession, it is crucial for dentistry to have an insight into the strong pressures exerted by the aesthetic expectations of the society in which we live and work and the potential consequences for people who do not conform (eg lower perceptions of intelligence, impacted job opportunities).^36^ As a result of oral health stigma and shame, people can experience loneliness, lower self-confidence and poorer-quality of life.^37^ These are strong motivators to seek appearance-enhancing procedures abroad, irrespective of the potential negative consequences:

- ‘This decision isn't an easy one for many, but if you see someone with a Turkey smile, please be kind and have understanding how hard and terrifying it was for those people to undergo this. It's no walk in the park' (woman who has had dentistry abroad, article 12).

### Future research

This article has highlighted that journalists and the wider public are sourcing and sharing health information about dental tourism from social media platforms. In other studies, cross-sectional analysis of healthcare messaging on TikTok has been undertaken; the protocols for these are readily available online.^38^ For example, studies have been undertaken to ascertain the #oralhealtheducation messaging of TikTok videos. Further to this review, we recommend further research, including content analysis of TikTok videos undertaken at time intervals, to understand how the dental tourism landscape is changing and how to monitor and manage access to health content that could have long-term repercussions for oral health.

## Conclusions

Dental tourism for cosmetic enhancement is a rapidly growing trend, specifically among younger people and social media users. In this article, we have identified the common issues described in the UK media and the attitudes conveyed about cosmetic dentistry through journalistic strategies. Social media viral health trends were a means of distributing health (dis/mis)information and played a part in informing people of the opportunities of dental treatment outside of the UK. Commonly, people were quoted as underplaying or ignoring short- and long-term risks of aggressive cosmetic procedures. Contrastingly, others chose to share their poor dental outcomes in a bid to discourage others. The UK dental profession strongly advocated for minimally invasive approaches and the risk of litigation discouraged treatment of people who required remedial care following dentistry abroad. The media conveyed an undertone of scorn and stigmatisation toward people who had had cosmetic dentistry abroad and amplified the perspectives of social media users.

## Data Availability

All primary newspaper articles used in this manuscript are available upon written request to the corresponding author.
